# Short sleep duration is associated with specific food intake increase among school-aged children in China: a national cross-sectional study

**DOI:** 10.1186/s12889-019-6739-8

**Published:** 2019-05-14

**Authors:** Muqing Cao, Yanna Zhu, Fan Sun, Jingyin Luo, Jin Jing

**Affiliations:** Department of Maternal and Child Health, Faculty of Public Health, Sun Yat-sen University, No. 74, Zhongshan 2nd Road, Yuexiu, Guangzhou, 510080 China

**Keywords:** Sleep duration, Food intake, Sugar beverage, Vegetable, Fruit

## Abstract

**Background:**

The relationship between sleep duration and food intake is unclear. This study aims to examine the relationship among children aged 6–17 years in China.

**Methods:**

The sample consisted of 70,519 children aged 6–17 years, which were randomly selected from 7 representative areas from China, from September to November, 2013. In the structured questionnaire, children reported daily sleep hours (less than 7 h, 7–9 h and more than 9 h), weekly food intake amount (including vegetables, fruit, sugar beverages and meat), physical activity and sedentary time. The relationship of sleep duration with vegetable, sugar beverage, fruit and meat intake was evaluated by multi-nominal logistic regression and multi-variable adjusted.

**Results:**

A total of 62,517 children (51.6% boys) completed the study. Short sleep duration (SSD, < 7 h) was independently associated with increased sugar beverage intake (SBI, Odd Ratio, OR: 1.29, 95% CI: 1.19–1.40) but decreased vegetable (VI, OR: 0.94, 95% CI: 0.90–0.98) & fruit intake (FI, OR: 0.94, 95% CI: 0.88–0.99). Stratified by age and gender, SSD increased SBI for boys of both young (6–12 years) & older (13–17 years) groups and older girls (ORs: 1.25, 1.25, 1.49, 95% CI: 1.08–1.44, 1.04–1.50, 1.22–1.81, respectively), but decreased VI and FI for older girls (ORs: 0.84& 0.81, 95% CI: 0.74–0.96& 0.68–0.96, respectively).

**Conclusions:**

Among school-aged children in China, short sleep duration was associated with increased risks of more sugar beverage intake among those younger and boys but less vegetable & fruit intake among those older and girls. Longitudinal research is needed to clarify the causation in between.

## Background

Sleep is an important modulator of growth, maturation, and health in children and adolescents [1]. However, the average sleep duration has been decreasing worldwide for decades [2] as a marker of modern society progress. The US Gallup polls affirm that modal sleep tended to last 8 h in 1979 compared with 6.6 h in 1998 [3]. As for children, a recent report from Sweden confirms a gradual decrease in the number of children who acquired the recommended sleep hours for children and adolescents [4]. Similar trends are reported in China and Australia [5, 6]. Thus, short sleep is, indeed, a global phenomenon.

Research corroborates that short sleep may lead to endocrine alternation [7], including the decreased levels of leptin, glucose tolerance, and insulin sensitivity and the increased levels of ghrelin, hunger, and appetite [8]. In addition, certain studies affirm a linkage between short sleep and specific behaviour changes, such as less physical activity, highly sedentary behaviour [7], and food intake [9]. This affirmation is based on energy balance hypothesis, which elucidates that short sleep causes additional energy expenditures, thereby causing the body to automatically reserve energy via reducing activity and increasing food intake [10].

A few studies [11–13] address short sleep and food intake among children, but the results affirm a discrepancy in the relationship between sleep duration and carbohydrate intake. As the experimental study observes that short sleep duration increased children’s fat and carbohydrate intake [11], an observational study among preschool children verifies that short sleep is linked to decreased carbohydrate intake [12]. On the one hand, the well-controlled experimental condition may hinder the generalization of the findings. Although several observational studies confirm a relationship between sleep duration and unhealthy food intake, the big age ranges from preschool children to adolescents and the followed natural sleep requirement decrease, along with more freedom of food choice [12–14] may hinder the generalizability of these conclusions.

Sleep duration generally decreases when children get old and reach adulthood, especially after puberty [15]. Thus, children with rapid growth may possibly be more affected by sleep duration compared with adults who have a relatively stable sleep patterns and physical conditions [16]. However, current epidemiologic studies usually focus on adults [17–19]. In this case, exploring the relationship between different diet intakes and sleep durations in a large representative child population is necessary. On the other hand, sleep duration and diet intake are gender- and age-related, but information on the two variables are limited. In addition, this association remains obscure in the Chinese children population, considering that Chinese children have different dietary habits with western children [20], and the sleep-diet change rapidly during children growth, this research with wide range of age groups is significantly urgent.

We aim to examine the relationship between different sleep durations (short, middle, and long) and food intakes among Chinese schoolchildren and further explore the gender- and age-dependent effect thereof. Given that human beings acquire different nutrients and potential health-related risks from different foods, we focused on four food groups that Chinese children usually consume daily [21, 22]. These are sugar beverages, meat, fruits, and vegetables. In addition, we also described the food intake per gender and age. We expected that short sleep duration would be related to high food intake with high energy density, such as sugar beverages, and that the association between sleep duration and food intake amount is age- and gender-dependent.

## Methods

### Participates enrollment

Seven research centers were chosen in China (located in Beijing, Tianjing, Liaoning, Ningxia, Shanghai, Changsha, and Guangzhou, respectively), for they represent the population for the northern (Beijing, Tianjing), northeastern (Liaoning), northwestern (Ningxia), eastern (Shanghai), central (Changsha), and southern (Guangzhou) parts of China [23]. The detail of the recruitment of children and the survey of baseline information for all the centers were described in our previous published protocol [17]. In total, there were 70,519 children aged 6–17 years agreed to take part into the national survey. The study was approved by the Sun Yat-sen University Ethics Committee, and all parents/guardians of children signed the informed consent.

### Anthropometric measurement

Anthropometric measurement was performed to all of children from September to November 2013. Height (centimeter, cm) and weight (kilogram, kg) were measured by qualified technicians in the corresponding school. Detail of measurement and body mass index (BMI) calculation could be found in our published article [6].

### Questionnaire survey

A standardised questionnaire that developed on the basis of the information, motivation, and behavioural skills model [24] was designed to collect demographic data (examination date, birth date, gender, education level of mother, and monthly household income), physical activity and sedentary lifestyle (weekly hours of high-level and middle-level physical activities, walking, and sedentary behaviours), and dietary intake (daily intake of meat, sugar beverages, fruits, and vegetables). Details of the questionnaire including social demographic information, daily food intake, daily physical activity/edentary behavior, sleep duration and quality control procedure have been described in our published article [6].

### Statistical analysis

Data were inputted using EpiData 3.0 software (EpiData Association, Odense, Denmark) and analyzed using Statistical Package for the Social Sciences (SPSS, version 22.0, IBM Corporation, New York). Descriptive statistics were calculated for all the variables, including continuous variables (presented as mean values± standard deviation or median with quartile) and categorical variables (presented as proportions). The differences of categorical variables were evaluated by chi-square tests. For continuous variables, t-tests (normal distribution) or Mann- Whitney U test (abnormal distribution) were used to evaluate gender differences and age group differences. The association between sleep duration (independent variable) and food intake (dependent variable) was evaluated by multi-nominal logistic regression models, which were initially adjusted for age and gender (model 1); intensive physical activity, moderate physical activity, walking, sedentary behaviour, mother’s educational level, and household income were subsequently introduced into the model (model 2). Cluster data from 7 research centers were also introduced into the model, and a robust standard error was used to minimize the effect that the data obtained from different areas may have. The results were reported by odds ratios (ORs) and corresponding 95% confidence intervals (CIs). The LSD group was considered as the reference group in the regression models. *p* values less than 0.05 were considered statistically significant.

## Results

### Baseline characteristics of the study population

A total of 8002 children were excluded because they did not provide essential information in their questionnaires (sleep duration or at least one type of food intake). The sample comprised 62,517 children. Their mean age was 10.82, and 48.4% were girls. Compared with the retained participants, those excluded from the analysis had no difference in age, the percentage of males, or baseline BMI. Table 1 describes the baseline characteristics of the participants. Compared with younger children (aged 6–12 years), older children (aged 13–17 years) had higher mean values of BMI, daily walking time, PC game time, and homework time (all *p* < 0.001) but lower mother’s educational level and household income (all *p* < 0.001). A total of 7.5% younger children and 37.1% older children reported SSD per night. In total, daily diet intakes servings were 0.41, 1.16, 1.28, and 1.79 (SBI, MI, FI, VI, respectively). Compared with the younger group, older children had higher daily SBI and meat intake (both *p* < 0.001) but lower mean values of FI and VI (both *p* < 0.05). Compared with girls, boys in the sample had higher BMI, IPA, MPA, walking, TV watching, PC game time, and household income but lower mother’s educational level (all *p* < 0.01). In addition, fewer boys reported SSD but had higher SBI, MI, and FI relative to girls (all *p* < 0.001).

**Table 1:** Sample characteristics across age and gender groups in the national school-based survey

	All (*n* = 62,517)	6–12 years old (*n* = 40,924)	13–18 years old (*n* = 20,593)	P Value for age groups	Boys (*n* = 32,257)	Girls (*n* = 30,260)	*P* Value for gender
Child characteristics, mean ± SD, median with quartile or %							
Female	48.4	47.3	50.5	< 0.001	0	100	< 0.001
Age (years)	10.82(3.30)	8.98(1.88)	14.64(1.37)	< 0.001	10.92(3.91)	11.06(3.22)	< 0.001
Height (cm)	145.82(17.09)	137.87(12.62)	163.50(8.41)	< 0.001	148.25(18.10)	145.57(14.84)	< 0.001
Weight (kg)	40.94(15.53)	34.50(11.49)	55.78(12.79)	< 0.001	43.71(17.17)	40.25(13.72)	< 0.001
BMI (kg^2^/m)	18.78(3.84)	17.71(3.42)	20.74(3.79)	< 0.001	19.13(4.03)	18.42(3.59)	< 0.001
Family income (%)				< 0.001			< 0.001
*< 5000 yuan/month*	28.0	27.0	29.9		27.1	29.0	
*> 5000 yuan/month*	14.2	15.2	12.3		14.0	14.4	
*Not known*	57.8	57.8	57.8		58.9	56.6	
Maternal educational level (%)				< 0.001			0.004
*< 9 years*	47.4	43.4	56.0		48.1	46.6	
*9–12 years*	26.2	27.0	24.4		25.9	26.5	
*> 12 years*	26.4	29.6	19.6		26.0	26.9	
Physical activity (Hours/day)							
IPA	0.21 (0.05, 0.57)	0.21 (0.02, 0.57)	0.23 (0.07, 0.57)	0.07	0.29 (0.07, 0.67)	0.19 (0.01, 0.48)	< 0.001
MPA	0.22 (0.00, 0.64)	0.23 (0.00, 0.64)	0.21 (0.00, 0.57)	0.21	0.25 (0.00, 0.67)	0.21 (0.00, 0.50)	< 0.001
Walking	0.48 (0.14, 1.00)	0.42 (0.00.14, 1)	0.50 (0.14, 1.00)	< 0.001	0.50 (0.14, 1.00)	0.43 (0.14, 1.00)	< 0.001
Sedentary behaviour (hours/week)							
TV watching	3.50 (1.75, 7.23)	5.25 (3.50, 8.17)	3.50(0.00, 7.00)	< 0.001	4.67 (2.33, 8.17)	3.50 (1.17, 7.00)	< 0.001
PC game	2.33 (0.00, 7.00)	2.33 (0.00, 7.00)	3.50 (0.00, 7.00)	< 0.001	3.50 (0.00, 7.00)	2.33 (0.00, 7.00)	< 0.001
Homework	12.25 (7.00, 17.50)	10.50 (7.00, 14.00)	15.75 (10.50, 21.00)	< 0.001	10.50 (7.00, 17.50)	14.00 (7.00, 18.00)	< 0.001
Sleep duration				< 0.001			< 0.001
*LSD*	25.3	35.0	6.7		26.5	24.1	
*MSD*	57.1	57.5	56.3		57.9	56.2	
*SSD*	17.6	7.5	37.1		15.6	19.7	
Diet intake (serves/week)							
*Sugar Beverage*	1 (0, 3)	1(0, 2)	0 (2, 5)	< 0.001	2 (0, 4)	1 (0, 2)	< 0.001
*Meat*	1 (1, 19)	7 (3, 8)	7 (3, 10)	< 0.001	7 (4, 12)	6 (3, 7)	< 0.001
*Fruits*	7 (4, 14)	7 (5, 14)	7 (3, 12)	< 0.001	7 (4, 12)	7 (4, 14)	< 0.001
*Vegetables*	10 (7, 14)	10 (7, 14)	10 (7, 14)	0.41	10 (7, 12)	10 (7, 14)	0.27

*BMI* Body mass index, *IPA* intensive physical activity, *MPA* moderate physical activity, *LSD* Long sleep duration, *MSD* Middle sleep duration, *SSD* Short sleep duration. Sample characteristics are presented as Mean ± Standard deviation (normally distributed, age, height, weight and BMI), Median with quartile (median, Q25 and Q75, including physical activity, sedentary behaviours and diet intake) and frequency (%, family income, maternal educational level and sleep duration)

**Fig. 1:**
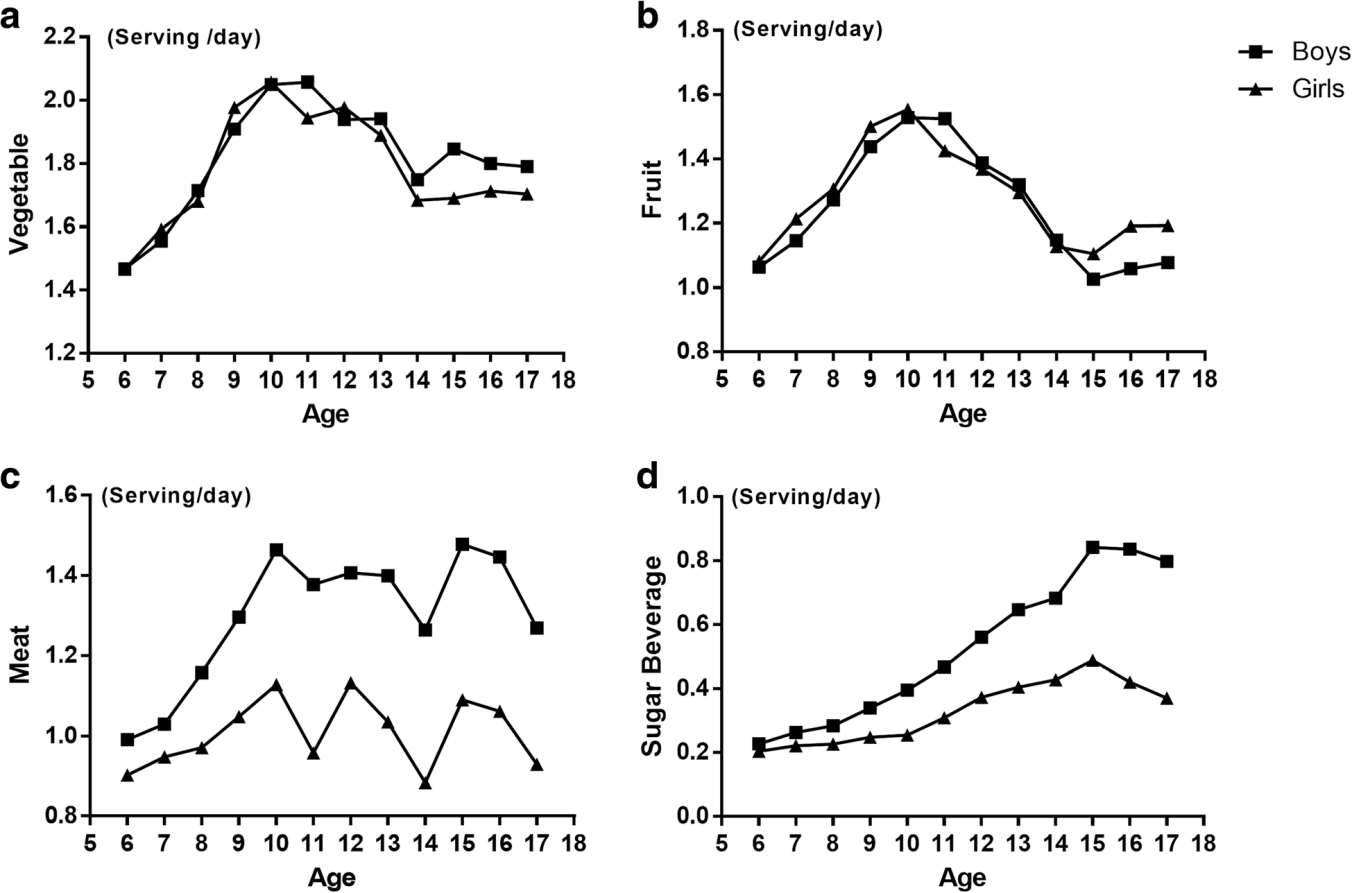
Diet intake inlcuding vegetable (**a**), fruit (**b**), meat (**c**) and sugar beverage (**d**) in different age among Chinese children aged 6–17 years (the y-axis is not in all instances including 0)

**Fig. 2:**
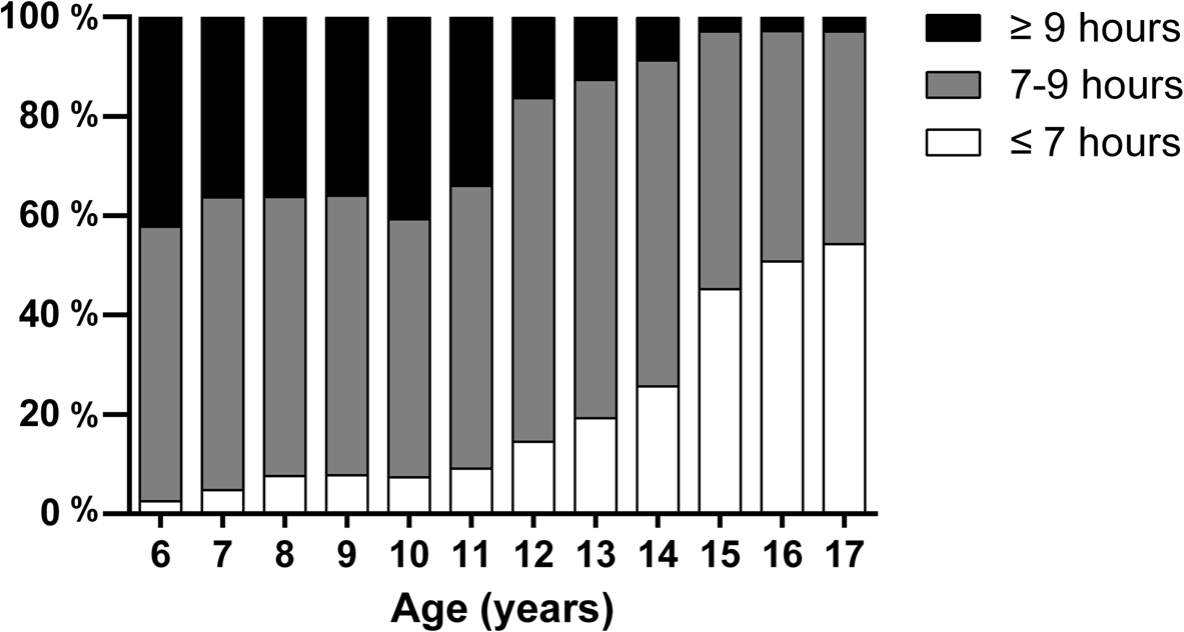
Distribution of sleep duration among Chinese children aged 6–17 years

Figure 1 depicts diet intakes curves by age and gender. VI and FI increased among children aged 6–10 years, decreased during 11–14 years and finally remained stable among children aged 15–17 years (Fig. 1a and b). Boys and girls showed a similar pattern. Children aged 6–10 years also had an increased MI amount, and a clear gender-specific effect can be observed (Fig. 1c). In addition, for children of all ages, boys had more meat intake than girls. SBI increased gradually during the age of 6–14 among boys (0.2–0.8 serves/ day) and girls (0.2–0.4 serves/ day) and then slightly decreased during the age of 15–17. The trend affirmed that children tend to have less sleep hours as they grow up (Fig. 2) and that gender-specific patterns are similar between boys and girls.

### Association between diet intake and sleep duration

Table 2 summarises the association between different volume of diet intakes and sleep duration. After age and gender are adjusted, per serve increase of SBI was associated with increased risk of MSD (OR: 1.08, 95%CI: 1.04–1.12) and SSD (1.20, 1.15–1.26), per serve of decrease of VI and FI were associated with increased risk of MSD (OR: 0.96 and 0.97, 95%CI: 0.94–0.98 and 0.95–0.99, VI and FI, respectively) and SSD (OR:0.91 and 0.91, 95%CI: 0.89–0.93 and 0.88–0.94) compared with the LSD group (Table 2). After physical activity/inactivity, education level of mother, and household income were adjusted, the analysis confirmed a similar trend (all *p* < 0.05, model 2, Table 2).

**Table 2:** Odds ratio (OR) and 95% confidence interval (CI) for middle and short sleep duration according to food intake among children aged 6–17 years in China

	LSD (*n* = 24,090)	MSD (*n* = 29,361)		SSD (*n* = 9066)	
		Model 1^a^	Model 2^b^	Model 1^a^	Model 2^b^
Sugar beverage	1	1.08(1.04–1.12)^*^	1.12(1.04–1.21)^*^	1.20(1.15–1.26)^*^	1.29(1.19–1.40)^*^
Vegetable	1	0.96(0.94–0.98)^*^	0.96(0.93–0.98)^*^	0.97(0.95–0.99)^*^	0.94(0.90–0.98)^*^
meat	1	0.99(0.97–1.01)	1.01(0.97–1.05)	0.97(0.95–1.00)	1.00(0.95–1.06)
Fruit	1	0.91(0.89–0.93)^*^	0.93(0.89–0.97)^*^	0.91(0.88–0.94)^*^	0.94(0.88–0.99)^*^

*LSD* Long sleep duration, *MSD* Middle sleep duration, *SSD* Short sleep duration

a. Adjusted for age, gender

b. Additionally adjusted for intensive physical activity, moderate physical activity, walking, sedentary behaviour, education level of mother and household income per month

^*^ P < 0.05, compared with children with LSD

After age was stratified, and multi-variable was adjusted, per serve increase of SBI was associated with increased risk of MSD (OR: 1.12, 95%CI: 1.03–1.22) and SSD (OR: 1.32, 95%CI: 1.18–1.47), per serve of decrease of VI was associated with increased risk of MSD (OR: 0.96, 95%CI: 0.93–0.99,) among children aged 6–12 years, compared with the LSD group (Table 3). As to those aged 13–17 years, compared with LSD group, per serve increase of SBI was associated with increased risk of SSD (OR: 1.25, 95CI: 1.07–1.46), per serve of decrease of FI were associated with increased risk of MSD (OR: 0.88, 95%CI: 0.80–0.97) and SSD (OR: 0.83, 95%CI: 0.74–0.93) (Table 3).

**Table 3:** Odds ratio (OR) and 95% confidence interval (CI) for middle and short sleep duration according to food intake among children aged 6–17 years in China (stratified by age and gender ^a^)

	LSD	MSD	SSD
Stratified by age			
6–12 years (n = 40,924)			
Sugar beverage	1	1.12(1.03–1.22)^*^	1.32(1.18–1.47)^*^
Vegetable	1	0.96(0.93–0.99)^*^	0.97(0.91–1.03)
meat	1	1.01(0.97–1.05)	0.99(0.93–1.07)
Fruit	1	0.95(0.91–0.99)	1.04(0.97–1.12)
13–17 years (*n* = 25,093)			
Sugar beverage	1	1.09(0.94–1.27)	1.25(1.07–1.46)^*^
Vegetable	1	0.95(0.88–1.03)	0.93(0.86–1.02)
meat	1	1.08(0.98–1.19)	1.09(0.98–1.21)
Fruit	1	0.88(0.80–0.97)^*^	0.83(0.74–0.93)^*^
Stratified by age & gender			
6–12 years			
Boys (*n* = 21,576)			
Sugar beverage	1	1.12(1.01–1.24)^*^	1.25(1.08–1.44)^*^
Vegetable	1	0.96(0.92–1.01)	0.99(0.92–1.08)
meat	1	1.00(0.95–1.05)	0.97(0.88–1.06)
Fruit	1	0.92(0.87–0.98)^*^	0.98(0.88–1.08)
Girls (*n* = 19,348)			
Sugar beverage	1	1.11(0.96–1.30)	1.49(1.22–1.81)^*^
Vegetable	1	0.96(0.92–1.01)	0.93(0.84–1.02)
meat	1	1.02(0.96–1.10)	1.05(0.94–1.18)
Fruit	1	0.98(0.92–1.05)	1.14(1.03–1.28)^*^
13–17 years			
Boys (*n* = 10,681)			
Sugar beverage	1	1.13(0.95–1.35)	1.25(1.04–1.50)^*^
Vegetable	1	1.03(0.93–1.14)	1.01(0.90–1.13)
meat	1	1.05(0.93–1.17)	1.04(0.91–1.18)
Fruit	1	0.91(0.79–1.03)	0.84(0.73–0.97)^*^
Girls (n = 10,912)			
Sugar beverage	1	1.05(0.79–1.39)	1.30(0.97–1.73)
Vegetable	1	0.86(0.77–0.97)^*^	0.84(0.74–0.96)^*^
meat	1	1.12(0.93–1.36)	1.17(0.95–1.43)
Fruit	1	0.85(0.73–1.00)	0.81(0.68–0.96)^*^

*LSD* Long sleep duration, *MSD* Middle sleep duration, *SSD* Short sleep duration

a. Adjusted for age, gender, physical activity/ inactivity, education level of mother and domestic income per month

* *P* < 0.05, compared with children with > 9 h sleep

We further stratified the dataset by gender. Among younger boys aged 6–12 years, increased risk of MSD and SSD were associated with per serve increase of SBI (OR: 1.12 and 1.25, 95%CI: 1.01–1.24 and 1.08–1.44, MSD and SSD, respectively) and increased risk of MSD was also associated with per serve decrease of FI (OR: 0.92, 95%CI: 0.87–0.98) (Table 3). As to young girls, increased risk of SSD were associated with per serve increase of SBI (OR: 1.49, 95%CI: 1.22–1.81) and VI (OR: 1.14, 95%CI: 1.03–1.28) (Table 3). For older boys aged 13–17 years, increased risk of SSD were associated with per serve increase of SBI (OR: 1.25, 95%CI: 1.04–1.50) but per serve decrease of VI (OR: 0.84, 95%CI: 0.73–0.97) (Table 3), as to girls with same age, increased risk of MSD and SSD were associated with per serve decrease of VI (OR: 0.86 and 0.84, 95%CI: 0.77–0.97 and 0.74–0.96; MSD and SSD, respectively) and FI (OR: 0.85 and 0.81, 95%CI: 0.73–1.00, 0.68–0.96; MSD and SSD, respectively) (Table 3).

## Discussion

Short sleep duration of children has become an international epidemic in recent years, and the sleep-related morbidity has increased in many countries [25]. Food intake alternation is believed to be linked to short sleep but has not been fully explored yet, particularly in the Chinese population. Using a national cross-sectional study with 62,517 children aged 6–17 years, we studied sleep duration’s role (independent variable) in food intake (dependent variable), and found that the short sleep duration (less than 7 h) prevalence was 17.6% and was higher in girls and older children. Importantly, SSD was related to the increased SBI among 6- to 12-year-old children and 13- to 17-year-old boys. For girls aged 13–17 years, FI and VI are positively associated with sleep duration. Generally, the results of this study validate that the association between short sleep duration and food intake is age- and gender-dependent. Insufficient sleep duration is associated with increased chances for drinking sugar beverages among younger children and boys, but decreased chances for eating vegetables and fruits among older children and girls.

Insufficient sleep is associated with the intake of food with high glycemic index (GI) [26]. On the basis of the hypothesis, short sleep leads to over energy expenditure [27]. Consequently, the body needs to recover energy rapidly, and high GI food increases blood glucose quickly. From another point of view, short sleep causes metabolic change, which increases ghrelin and decreased leptin secretion [8]. Experimental studies corroborate that metabolic change caused by sleep deprivation results in several hedonic food choices [28], which may also explain the increased high GI food, such as sugar beverage. In our study, we also affirmed an association between increased SBI and SSD/MSD. Although the relationship is cross-sectional, the association trend is in agreement with the hypothesis that the longitudinal study tested [29].

Our results complied with previous research that asserts that VI and FI are negatively associated with insufficient sleep [13], but the mechanism is still unclear. One hypothesis on hedonic eating behaviour after short sleep [30] is that children tend to eat unhealthy food and avoid healthy food because the latter provides far less energy density per unit volume [31]. This affirmation makes evolutionary sense as food with high energy density supported human survival in ancient times. In this case, this type of food could be a protective mechanism as avoiding vegetables and fruits can save limited stomach volume for high energy food because short sleep itself could be treated as stress [32]. Another potential mechanism indicates that lack of sleep leads to considerable eating in general. In addition, the types of food available to the youth late at night are likely to be convenience items, such as fast food, as opposed to vegetables that typically require fair preparation and are eaten with meals [13].

Although numerous studies reported a sleep-diet relationship, the age dependent effect was seldom addressed [12, 14, 33, 34]. Considering that the OR values are relatively small, stratified analysis had been made in order to decrease the potential co-founding effect of age and gender (Table 3). Our data reported that food intake and sleep duration are affected by age (Figs. 1 and 2). Sleep duration declined, and the food intake pattern changed during puberty. Thus, we performed the analysis using stratified data by age groups, and took the reported puberty on-set time of Chinese children (12 years old) [35] as a cut-off point. Although the recommended sleep duration for children is 9 h, certain studies corroborate that sleep decline is acceptable during puberty [36]. In our study, children aged 6–12 years have increased SBI at MSD and SSD (Table 3), but, for those aged 13–17 years, MSD is no longer associated with more SBI. This finding showed that MSD might be acceptable for older children and does not necessarily induce energy reserve, whereas SSD is still too short for them. Moreover, we confirmed that the less FI associated with insufficient sleep happened only amongst older children. Figure 1b illustrates that FI increased by age before puberty and had a dramatic decline after 11 years old, which was paralleled with a sleep duration decrease (Fig. 2). Thus, an insufficient sleep–fruit relationship could have a dose-dependent response. From another point of view, although fruit is usually classified as healthy, several types of fruits, such as watermelon, have high GI [37]. Specific fruit species in this research were not addressed, so the different association between short sleep and age groups is ascribed to discrepancy on the fruit preference in between, which may attributed to the liberty of food choice increased as child grow up [38].

Compared with boys, girls had a different pattern of food intake, especially after puberty (Fig. 1). Our results showed that girls aged 6–12 years with SSD showed more FI (Table 3). Although no current reports related to fruit preference depending on age are made, girls aged 6–12 years possibly took high GI fruits as a replacement for sugar beverage, for they may have specific food preference relative to boys [39]. Apart from FI, the association between sleep duration and SBI was gradually weak from younger to older children and boys to girls (Table 3). This finding may be because older children need less sleep, and girls further require shorter sleep durations relative to boys of the same age [40]. Interestingly, although the association between sleep duration and SBI weakens by age, its association with VI is strengthened. After age and gender are stratified, only girls aged 13–17 years had less VI when sleep was less than 9 h (Table 3). Sleep duration decrease would lead to total energy intake growth [8]. However, we did not find an increased chance of SBI, FI, and MI among these girls. In our study, other food types, such as rice and wheaten food that Chinese people eat most, were not included. Thus, we cannot conclude that children may increase rice and wheaten food intake as a replacement.

No study has currently reported the association between sleep duration and meat intake. Meat is considered protein rich, and certain studies confirm that short sleep may decrease the need for protein intake [41]. However, MI did not change in each age and gender group in our study. The MI level is relatively low in the Chinese population compared with that of Western countries [42]. Alternatively, the main source of dietary protein in China is usually soy products [43]. Thus, the short sleep effect on protein intake may reflect not on MI but on soy food instead. Another potential mechanism is the fat intake increase on short sleepers [44]. Thus, the short sleep effect on protein intake may reflect not on MI but on soy food instead. As meat is a main source of fat intake, the effect of a decrease in protein could be buffered by an increased intake of fats, which may also explain the unchanged MI in our study.

Certain studies affirm the association between sleep and diet intake, especially because SBI maybe bidirectional [45]. Although no study has reported that sugar leads to a sleep decline before, carbohydrate intake may decrease the overall sleep quality with sleep architecture modification [33], which leads to the decline of sleep duration. Although we did not address total carbohydrates in our research, sugar in beverages possibly has a similar effect as carbohydrates, leading to a sleep decline. In addition, glucose fluctuation caused by sugar intake may affect the emotional state [46], and the latter is associated with sleep quality, which can also be a potential mechanism.

The potential relationship between sleep and food intake is also worthy of note. High-quality diet and adequate sleep duration represent a relatively healthy lifestyle [47]. In this case, vegetable and fruit intake can merely accompany long sleep duration rather than be the cause of it. In the meantime, sugar beverages are thought to be unhealthy, thereby possibly leading to short sleep duration. On the other hand, both sleep duration and diet choice are social/ psychological factor dependent and could be both consequence of one common factor. For instance, adolescents often detach from parents’ advice [48], especially those for health, this could be the reason of short sleep and unhealthy food choice.

Several limitations are found in this study. Given that the study was conducted in a cross-sectional manner, the causal pathways underlying the observed relationships could not be detected. The questionnaire has not been statistically validated nor tested for reliability. Sleep duration was reported by the parents of children under nine years old, which could occasionally be an ideal sleep duration that the parents think their children have rather than the latter’s actual sleep duration [49]. Children older than nine years personally reported the information, which may have led to self-reported bias. Missing of family income may cause information bias. An objective technique is considerably useful in conducting sleep research and generally deemed to be the optimal manner. In addition, our sleep duration category is slightly different from the recommendation of the National Sleep Foundation [50], which recommends a short sleep duration (8–10 h) among children aged over 14 years.

## Conclusion

This research described the relationship between sleep duration and food intake amongst Chinese children. Short sleep duration is associated with increased sugar beverage intake among those younger and boys, but decreased vegetable and fruit intake among those older and girls. Longitudinal research is necessary to clarify the causation in between, as well as the gender- and age-specific effect. In addition, studies that focus on a wider range of food species are also necessary.

## Abbreviations

FI: Fruit intake
LSD: Long sleep duration
MI: Meat intake
MSD: Middle sleep duration
SBI: Sugar beverage intake
SSD: Short sleep duration
VI: Vegetable intake
